# An unusual initial presentation of mantle cell lymphoma arising from the lymphoid stroma of warthin tumor

**DOI:** 10.1186/s13000-015-0444-4

**Published:** 2015-12-03

**Authors:** Ramir S. Arcega, Aaron J. Feinstein, Sunita Bhuta, Keith E. Blackwell, Nagesh P. Rao, Sheeja T. Pullarkat

**Affiliations:** Department of Pathology and Laboratory Medicine, UCLA David Geffen School of Medicine, UCLA Path & Lab Med, AL-134 CHS, BOX 951732, Los Angeles, CA 90095-1732 USA; Department of Head and Neck Surgery, UCLA David Geffen School of Medicine, Los Angeles, USA

**Keywords:** Mantle cell lymphoma, Warthin tumor, Collision tumor

## Abstract

**Background:**

Warthin tumors presenting concomitantly with a lymphoma is vanishingly rare with only 15 reported cases in English literature. Herein, we report an unusual initial presentation of a mantle cell lymphoma involving the lymphoid stroma of a Warthin tumor.

**Case presentation:**

A seventy-seven year old otherwise healthy gentleman with a 50-pack year smoking history presents with a slowly enlarging left cheek mass. CT scan of the neck demonstrated a left parotid gland tumor measuring 3.4 cm in greatest dimension. He underwent a left superficial parotidectomy, with subsequent histopathologic examination revealing a Warthin tumor with extensive expansion of the lymphoid stroma. Flow cytometric, immunohistochemical, and cytogenetic studies of the stromal component of the tumor confirmed the presence of a mantle cell lymphoma. Clinical staging demonstrated stage IVa disease, and was considered to be at low to intermediate risk due to the slow growth of the parotid lesion. The patient is undergoing close follow up with repeat PET-CT scans at six months.

**Conclusion:**

To the best of our knowledge, this is the first well documented collision tumor between mantle cell lymphoma and a Warthin tumor. This case also brings to light the significance of thorough evaluation of the lymphoid component of Warthin tumor.

## Background

Warthin tumor is the second most common benign tumor of the salivary gland [1]. The tumor is characterized by an epithelial component composed mainly of tall bilayered oncocytic columnar cells embedded within a lymphoid stroma [1, 2]. Lymphoma involving the stromal component is extremely rare, with the majority of those that have been described are in the setting of a widely disseminated lymphoma. Herein we describe a case of mantle cell lymphoma that primarily presented as part of the Warthin Tumor (i.e., a collision tumor). Karyotype studies detected the characteristic t(11;14)(q13;q32) translocation as part of a complex karyotype, and corroborated by immunohistochemistry studies. This case emphasizes the importance of a thorough evaluation of the lymphoid stroma of a Warthin Tumor, since the lymphoma cells can be easily confused with a normal reactive lymphoid component within the tumor.

## Case presentation

**Fig. 1:**
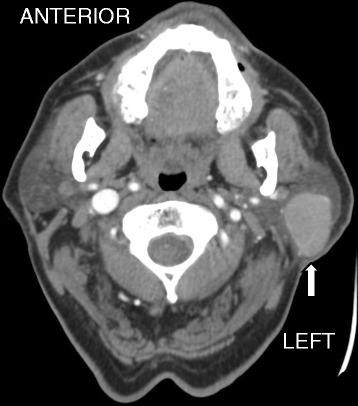
Computed tomography of neck soft tissue demonstrates an enhancing mass (white arrow) within the left parotid gland measuring 3.3 cm (anterior-posterior) × 2.2 cm (transverse) × 3.4 cm (craniocaudal). No enlarged lymph nodes or abnormal fluid collections are seen

A 70 year old otherwise healthy male patient with a 50-pack year smoking history who quit approximately 3 years ago, presented with a slowly enlarging left cheek mass. He first noted the lesion approximately 1 year prior to presentation. The patient reported undergoing a fine needle aspiration at an outside facility at which time some fluid was drained from the cheek mass, but the mass later re-accumulated. Over time he developed mild local pain and ipsilateral left ear discomfort. Contrast enhanced CT-scan of the neck demonstrated a left parotid gland tumor measuring 3.3 cm (anterior-posterior) × 2.2 cm (transverse) × 3.4 cm (craniocaudal) without pathologic cervical adenopathy (Fig. 1). The facial nerve function was intact, with a House-Brackmann score of 1 out of 6. The patient underwent a left superficial parotidectomy with a left sternocleidomastoid muscle flap reconstruction. He was discharged home on postoperative day 2 following an uneventful hospital course. Facial nerve function was full postoperatively. A complete blood count was within normal range at the time of diagnosis.

## Pathologic findings

**Fig. 2:**
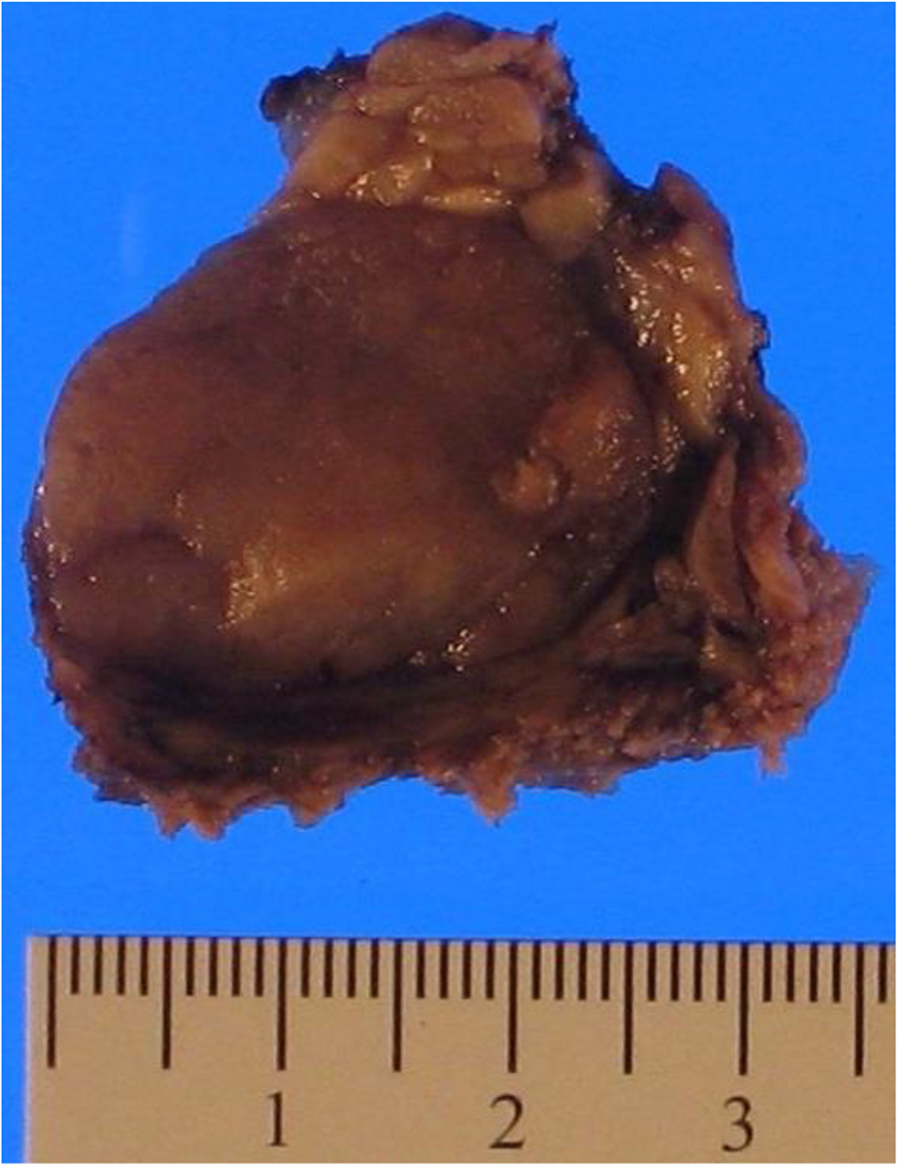
Gross photograph of a cut section of the parotid gland demonstrating a well circumscribed, homogeneous, tan lesion surrounded by a rim of normal salivary gland parenchyma (scale shown)

Gross examination of the left superficial parotidectomy specimen demonstrated a tan-red ovoid specimen with a lobulated external surface that measured 5.2 × 4.2 × 3.8 cm. Sectioning revealed a single well-circumscribed homogeneous tan mass measuring 4.1 × 2.2 × 2.1 cm (Fig. 2). Cut surfaces of the lesion had a homogeneous tan “fish-flesh” appearance. The uninvolved parenchyma was tan-red and lobulated.

**Fig. 3:**
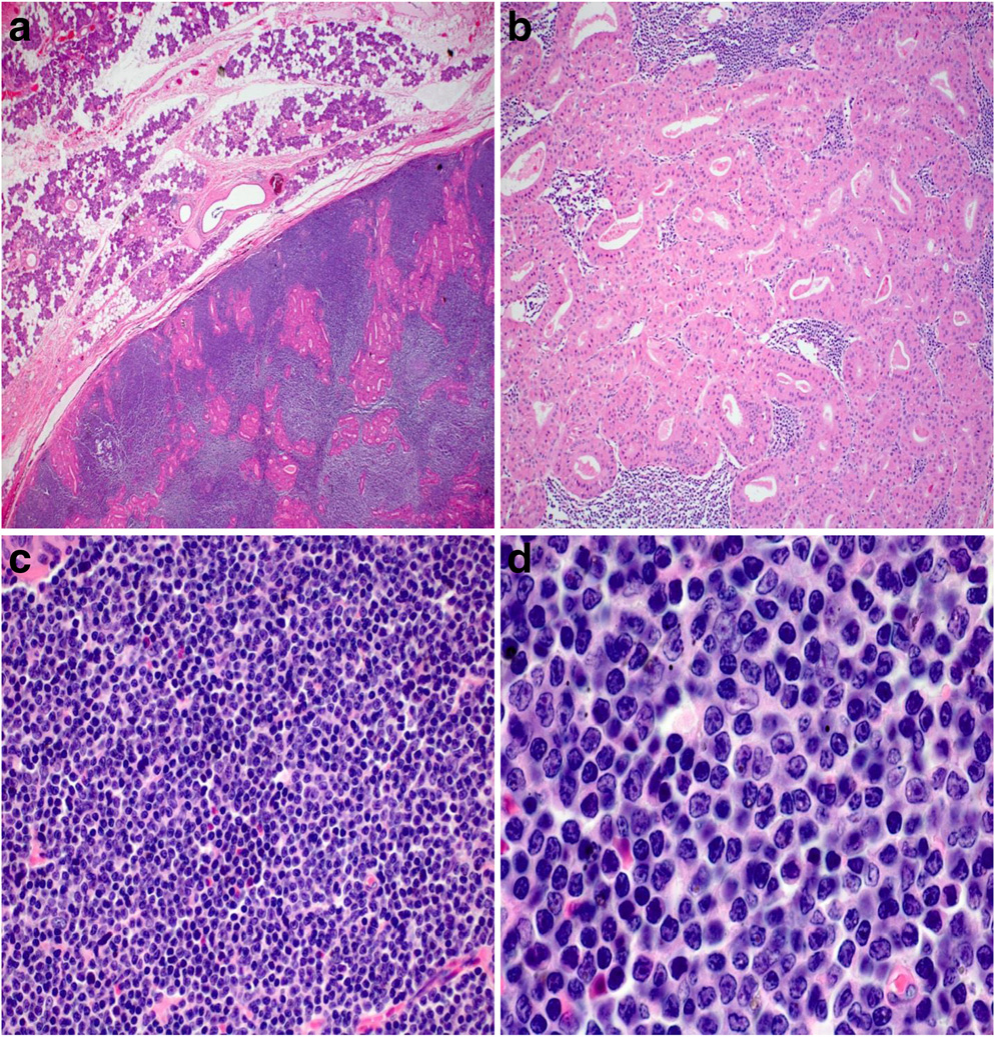
Mantle cell lymphoma arising from Warthin tumor stroma. **a** Histologic sections of the parotid gland demonstrate a well circumscribed lesion with a thin fibrous capsule that is sharply delineated from the adjacent uninvolved salivary gland parenchyma, with an expanded lymphoid stroma showing rare secondary follicular structures. **b** Higher magnification demonstrates the characteristic dual layered oncocytic cells of Warthin tumor. **c** The stromal lymphocytes are small to medium in size, with higher magnification (**d**) demonstrating clumped to vesicular chromatin patterns, irregular nuclear contours, inconspicuous to small distinct nucleoli, and scant clear cytoplasm. Hemotoxylin-eosin stain, original magnification 20× (**a**) 200× (**b**), 400× (**c**), and 1000× (**d**)

**Fig. 4:**
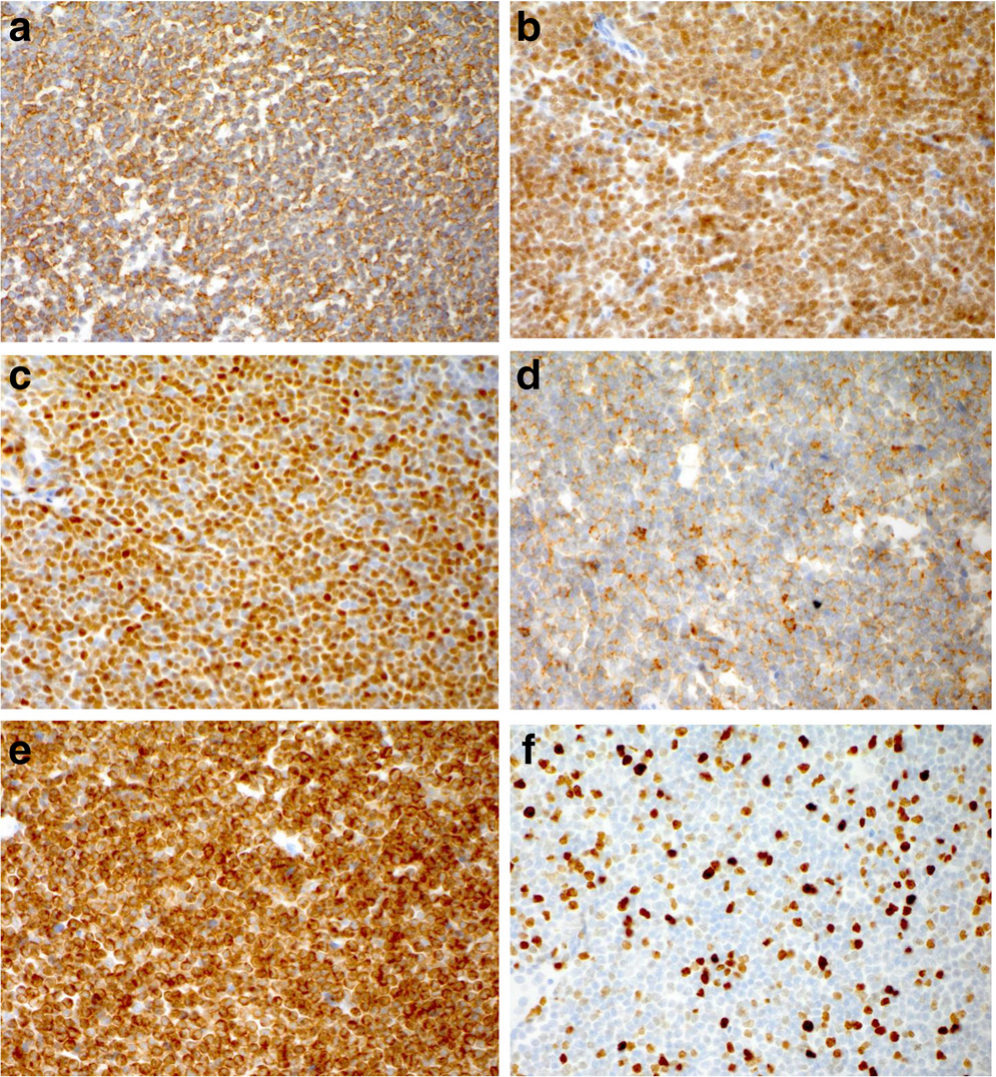
Immunohistochemical stains demonstrate the neoplastic lymphoid cells positive for **a** CD20, **b** PAX5, **c** Cyclin D1, **d** CD5, weak, **e** and BCL2. The Ki67 proliferation index (F) was estimated at 40 %. Immunohistochemical stain, original magnification,400× (**a**–**f**)

Microscopic examination revealed an intraparenchymal lesion composed of both epithelial and lymphoid elements encased within a thin fibrous capsule, sharply demarcated from the adjacent uninvolved salivary gland parenchyma (Fig. 3a). The epithelial component was composed of a double layer of tall columnar oncocytic cells with bland cytologic features arranged in a vague papillary and rare cystic architecture, both of which were compressed by the markedly expanded lymphoid stroma with rare secondary follicles seen (Fig. 3b). The lymphoid stroma was composed of small to medium sized lymphocytes with clumped to vesicular chromatin pattern, irregular nuclear contours, indistinct nucleoli, and scant cytoplasm (Fig. 3c and d). Mitoses and necrosis were inconspicuous. Lymphoepithelial lesions were not identified. Immunohistochemical stains showed the neoplastic cells positive for CD5 (weak), CD20, PAX5, Cyclin D1, BCL2, and negative for CD10, CD23, and BCL6 (Fig. 4a–e). The Ki67 proliferation index was estimated at approximately 40 % (Fig. 4f).

**Fig. 5:**
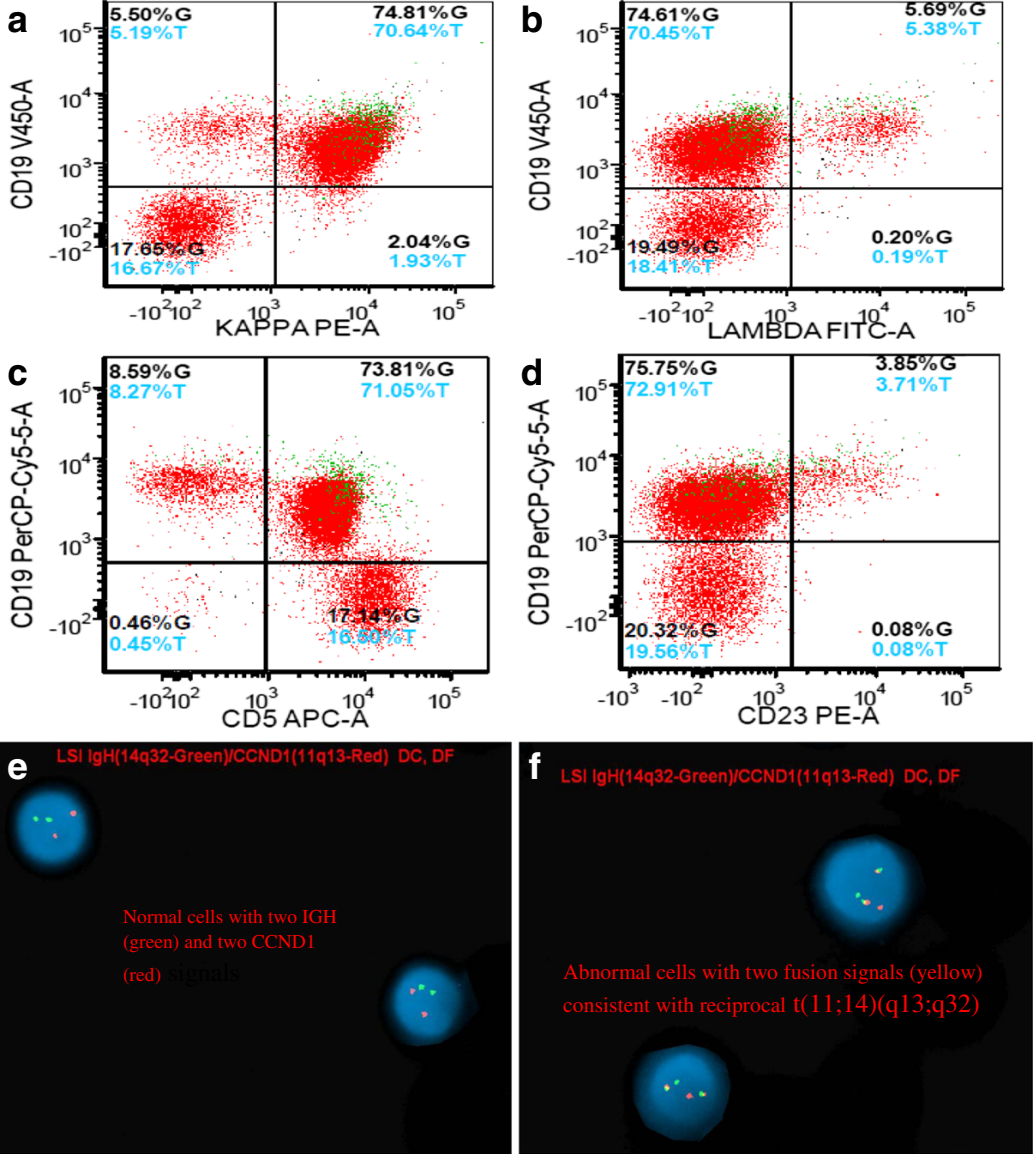
Flow cytometry performed on the specimen demonstrate a kappa-restricted B-cell population (**a**) and (**b**) co-expressing (**c**) CD5 and (**d**) are negative for CD23. **e** FISH studies using CCND1 (11q13 red); IGH (14q32 green) dual color-dual fusion probes - normal control, **f** FISH studies with lymphoma cells showing CCND1-IGH (yellow) fusion signal

**Fig. 6:**
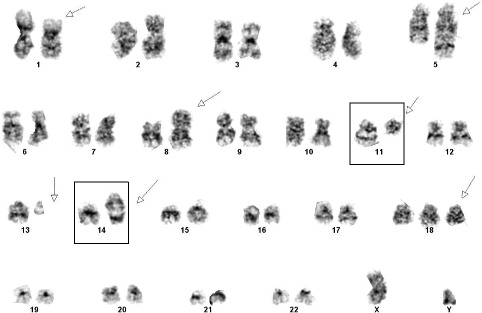
Chromosomal analysis showing t(11;14) [involved chromosomes in box] in association with a complex karyotype. Arrows highlight chromosome abnormalities (see text)

Flow cytometric studies of the mass demonstrated a kappa-restricted B-cell population co-expressing CD20, CD5 (dim), PAX5, FMC7, CD19, and negative for CD10 and CD23 (Fig. 5a–d). Flow cytometry was not performed on peripheral blood. The karyotype studies from cultured cells revealed a complex karyotype in 2 of 20 cells analyzed: 47,XY,del(1)(p36.1),ins(5;13)(q35;q12q32),add(8)(p21),t(11;14)(q13;q32),+18[2]/46,XY[19] with the characteristic t(11;14) translocation (Fig. 6). FISH analysis with the dual-color dual fusion IGH-CCND1 probes identified dual fusion signals (yellow) confirming the presence of a reciprocal t(11;14)(q13;q32) (Fig. 5e–f). Both karyotype and FISH studies were performed on the tissue mass.

## Discussion

After a thorough review of the English literature we conclude that this is the first case of a mantle cell lymphoma presenting within the lymphoid stroma of a Warthin tumor that was diagnosed at the time of histopathological assessment of a Warthin tumor excision. We were able to locate other case reports of non-Hodgkin lymphomas within Warthin tumor (15 cases, Table 1) [3–15] with eleven follicular lymphomas [3–7, 10–13], two chronic lymphocytic leukemia/small lymphocytic leukemia [9, 14], one Hodgkin lymphoma [8], and one T-Lymphoblastic Lymphoma [15]. A report from 1980 described a mantle cell lymphoma (formerly known as malignant centrocytic lymphoma) arising within a parotid gland with chronic myoepithelial parotitis, with an adjacent 0.5 cm Warthin tumor (cystadenolymphoma) as a secondary finding, but the lymphoma was not described to arise from within the lymphoid stroma of the tumor [16] as seen in the current case. In addition, since the reported case was diagnosed in 1980, the diagnosis was based on morphology alone, with no immunophenotypic support or establishment of t(11;14)(q13; q32).

**Table 1:** Summary of 15 cases of non-Hodgkin lymphoma involving Warthin tumor

Author (year)	Sex	Age	Location	Lymphoma
Colby and Dorfman (1979)	NA	NA	Parotid	FL
Miller (1982)	M	49	Right Mandible	FL
Hall (1985)	M	64	Right Parotid	FL
Banik (1985)	M	75	Left Parotid	FL
	M	76	Right Parotid	FL
Griesser (1986)	F	64	Palate	FL
Melato (1986)	M	69	Right Parotid	Hodgkin Lymphoma
Bunker and Locker (1989)	F	63	Left Parotid	SLL/CLL
Giardini and Mastore (1990)	M	57	Left and Right Parotid	FL
Medeiros (1990)	M	71	Left Parotid	FL
Shikhani (1993)	M	56	Right Parotid	FL
Park (2000)	F	68	Right Parotid	FL
	M	55	Right Parotid	FL
Saxena (2005)	M	60	Left Parotid	SLL/CLL
Giaslakiotis (2009)	M	81	Right Parotid	T-LBL

*FL* Folliclar Lymphoma

*SLL/CLL* Small Lymphocytic Lymphoma/Chronic Lymphocytic Leukemia

*T-LBL* T-Lymphoblastic Lymphoma

Warthin tumors are the second most common benign salivary gland tumor, with an average age at presentation of 62 years old, and have rarely been described before the age of 40 [1]. A strong association between smoking and Warthin tumors has been described, with an estimated incidence of eight times compared to that of nonsmokers [17]. The male to female ratio was 10:1 in 1953, whereas it was 1.2:1 in 1996 [1], which parallels the larger number of female smokers during this period [2]. Despite quitting smoking a few years prior to his presentation, our patient had an established 50-pack year smoking history. The patient denied other possible risk factors that have been linked to Warthin tumors, such as radiation exposure and autoimmune disorder.

Biologically it is quite feasible that lymphomas may arise within a Warthin tumor, since the lymphoid stroma is part of the systemic lymphoid tissue, and hence may be involved in disseminated lymphomatous involvement. Of the reported cases of lymphomas within Warthin tumors, the majority of patients either already had an established diagnosis at presentation, or systemic involvement was discovered at staging after initial diagnosis in the Warthin tumor.

Although patients with mantle cell lymphoma have a median survival of 3–5 years, with the vast majority of these patients not cured despite treatment [18], the prognosis of those which arise from within the confines of another tumor is virtually unknown. The most consistent histopathologic prognostic parameter is a high mitotic rate, which is defined as 10–37.5/15 hpf, with a high proportion of Ki67 positive cells (>40 % to 60 %) also an adverse prognostic indicator [18]. Blastoid and pleomorphic morphology, trisomy 12, and karyotypic complexity(≥ three or more chromosomal aberrations), have all been reported to have adverse prognostic features [18]. Despite the lack of observed mitotic activity in the current case, the Ki67 proliferative index was estimated to be 40 %. Blastoid and pleomorphic morphology was not evident. Although gains of chromosome 3q and deletions of 9q, both associated with poor prognosis were not seen in the current case, a complex karyotype was observed.

A thorough clinical staging is warranted in patients with mantle cell lymphoma, since most patients present with stage III or IV disease [18]. The patient was referred to his local medical oncologist. He had whole body diagnostic and contrast-enhanced CT imaging followed by FDG administration and PET image acquisition, which disclosed hypermetabolism in the left parotid area and a single liver lesion, likely representing metastatic disease, without other adenopathy. Bone marrow biopsy revealed 2 % involvement by B-cell lymphoma. He was staged as Stage IVa. Patient was considered to be at low-intermediate risk due to the slow growth of the parotid lesion, and elected to undergo close observation with plans for repeat PET-CT at 6 months.

## Conclusion

In summary, initial presentation of a malignant lymphoma within the lymphoid stroma of a Warthin tumor is extremely rare. This is the first reported case of a collision tumor presenting with mantle cell lymphoma and Warthin tumor. Emphasis must be made for a thorough examination of the lymphoid component in a Warthin tumor as clearly exemplified by our case.

## Consent

Written informed consent was obtained from the patient for publication of this Case Report and is available upon request.
